# Thoracic paravertebral block for perioperative lung preservation during VATS pulmonary surgery: study protocol of a randomized clinical trial

**DOI:** 10.1186/s13063-023-07826-8

**Published:** 2024-01-22

**Authors:** Jiayu Zhu, Biyu Wei, Lili Wu, He Li, Yi Zhang, Jinfeng Lu, Shaofei Su, Chunhua Xi, Wei Liu, Guyan Wang

**Affiliations:** 1 Department of Anaesthesiology, Beijing Tongren Hospital, Capital Medical University, Beijing, 100730 China; 2 Department of Anaesthesiology, Beijing Chest Hospital, Capital Medical University, Beijing, 101100 China; 3 Department of Anaesthesiology, Beijing Renhe Hospital, Beijing, 102600 China; 4 Central Laboratory, Beijing Obstetrics and Gynecology Hospital, Capital Medical University, Chaoyang, Beijing, 100026 China

**Keywords:** Postoperative pulmonary complications, Thoracic paravertebral block, Postoperative analgesia, Video-assisted thoracoscopic surgery

## Abstract

**Background:**

Postoperative pulmonary complications (PPCs) extend the length of stay of patients and increase the perioperative mortality rate after video-assisted thoracoscopic (VATS) pulmonary surgery. Thoracic paravertebral block (TPVB) provides effective analgesia after VATS surgery; however, little is known about the effect of TPVB on the incidence of PPCs. The aim of this study is to determine whether TPVB combined with GA causes fewer PPCs and provides better perioperative lung protection in patients undergoing VATS pulmonary surgery than simple general anaesthesia.

**Methods:**

A total of 302 patients undergoing VATS pulmonary surgery will be randomly divided into two groups: the paravertebral block group (PV group) and the control group (C group). Patients in the PV group will receive TPVB: 15 ml of 0.5% ropivacaine will be administered to the T4 and T7 thoracic paravertebral spaces before general anaesthesia induction. Patients in the C group will not undergo the intervention. Both groups of patients will be subjected to a protective ventilation strategy during the operation. Perioperative protective mechanical ventilation and standard fluid management will be applied in both groups. Patient-controlled intravenous analgesia is used for postoperative analgesia. The primary endpoint is a composite outcome of PPCs within 7 days after surgery. Secondary endpoints include blood gas analysis, postoperative lung ultrasound score, NRS score, QoR-15 score, hospitalization-related indicators and long-term prognosis indicators.

**Discussion:**

This study will better evaluate the impact of TPVB on the incidence of PPCs and the long-term prognosis in patients undergoing VATS lobectomy/segmentectomy. The results may provide clinical evidence for optimizing perioperative lung protection strategies.

**Trial registration:**

ClinicalTrials.govNCT05922449. Registered on June 25, 2023.

**Supplementary Information:**

The online version contains supplementary material available at 10.1186/s13063-023-07826-8.

## Introduction

Surgical resection is still the go-to treatment for lung cancer, which is the leading cause of cancer death [1]. As a minimally invasive operation, video-assisted thoracoscopic surgery (VATS) causes significantly less surgical trauma and systemic inflammation [2, 3] and has become the standard treatment method for lung cancer [4].

Postoperative pulmonary complications (PPCs) are one of the most common complications that occur after thoracoscopic lung cancer surgery, with an incidence of 40.8 %[5]. PPCs increase the hospitalization time, hospitalization cost, and perioperative mortality rate and affect the treatment effect and utilization of medical resources. One of the most pressing clinical issues is the development of measures to lower the prevalence of PPCs. Previous research has shown that lung protective ventilation strategies, including low tidal volume, positive end-expiratory pressure (PEEP), and low inhalation oxygen concentration, improve the prognosis of patients with lung injury, but they may not fully prevent acute lung injury caused by one-lung ventilation (OLV) during VATS [6].

The incidence of pain 24 h after VATS was 38% [7], and the incidence of chronic pain 6 months after VATS was 25% [8]. Poor postoperative analgesia will affect postoperative recovery, which may increase the risk of pulmonary complications due to insufficient respiratory function and weak sputum excretion [9, 10]. Thus, it is crucial to effectively control acute pain following VATS. Thoracic epidural anaesthetics (TEAs) have been shown in studies to help high-risk patients experience fewer respiratory complications [11, 12]. However, the high failure rate and incidence of complications limit its clinical application [9].

Ultrasound-guided thoracic paravertebral block (TPVB) is a regional block technique commonly used in thoracic surgery. Local anaesthetics can be injected into the paravertebral space to block the ipsilateral sympathetic and somatosensory nerves. TPVB combined with general anaesthesia (GA) can reduce pain after VATS, decrease the expression of matrix metalloproteinase-9, reduce the inflammatory reaction after thoracic surgery, improve the postoperative survival rate by blocking the unilateral sympathetic nerve [13], improve the postoperative rehabilitation of patients after VATS lung cancer radical surgery [14], and reduce the incidence of postoperative tumour recurrence [15]. According to a recent retrospective propensity score matching analysis [16], TPVB and GA together were linked to a decreased incidence of PPCs (29.8% vs. 34.2%). However, a prospective study on the effects of GA alone vs. GA coupled with TPVB on the incidence of PPCs following VATS pulmonary surgery has not been performed.

## Methods

### Objectives of the study

The aim of this study is to explore whether GA combined with TPVB can more effectively reduce the incidences of atelectasis, lung inflammation, and lung injury than GA alone during VATS pulmonary surgery to ultimately reduce the incidence of PPCs, protect the lungs, and improve the long-term prognosis of patients.

### Study design

**Fig. 1:**
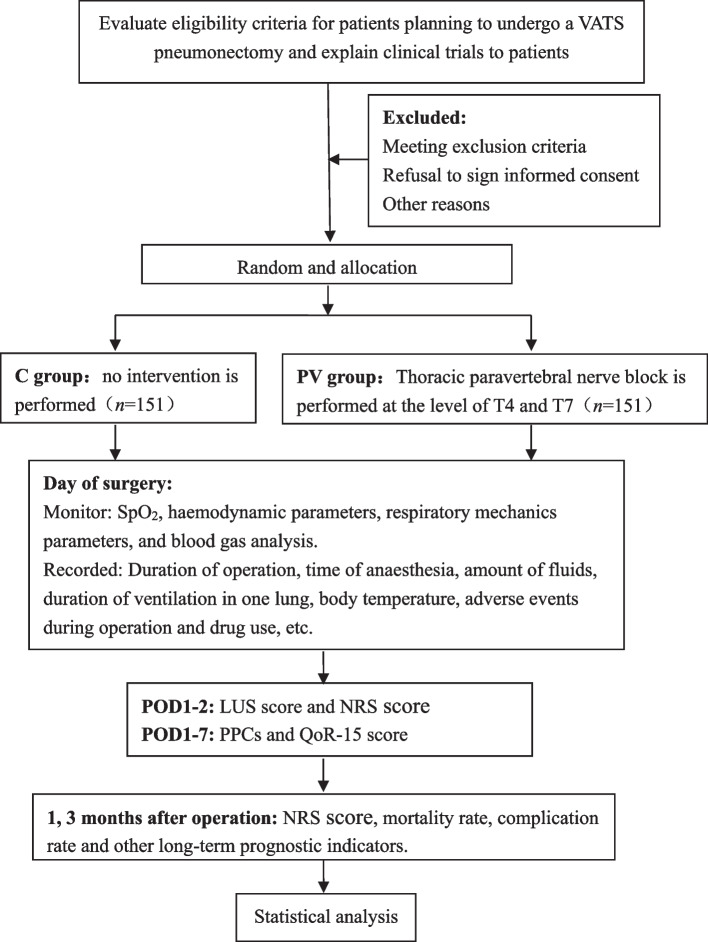
CONSORT flow diagram. VATS, video-assisted thoracoscopic; SpO2, pulse oxygen saturation; LUS, lung ultrasound; POD, postoperative day; NRS, numeric rating scale; QoR-15, quality of recovery with 15 items

This trial is an investigator-initiated, prospective, double-centre, randomized controlled trial. The Institutional Ethical Committee of Beijing Tongren Hospital (TREC2023-KY020) and The Institutional Ethical Committee of Beijing Chest Hospital ([2023] LS-KY-No.18) approved version 2.0 of the proposal on March 16, 2023, and June 16, 2023. This study has been registered with ClinicalTrials. gov (NCT05922449, Principal Investigator: Guyan Wang, https://clinicaltrials.gov/study/NCT05922449) and will strictly comply with the Good Clinical Practice guidelines and the Declaration of Helsinki. Any revisions to the plan or the informed consent form will be sent for approval by the ethics committee. If necessary, additional consent from the subject will be obtained. The study participants will be divided into two groups at a 1:1 ratio: the paravertebral block group (PV group, *n*=151; GA combined with TPVB) and the control group (C group, *n*=151; GA only). The specific flowchart is shown in the Consolidated Standards of Reporting Trials [CONSORT] diagram, Fig. 1. The (SPIRIT) 2013 Checklist is listed in Additional file 1.

### Participants and recruitment

The study will be conducted at Beijing Tongren Hospital, Capital Medical University, Beijing, China and Beijing Chest Hospital, Capital Medical University, Beijing, China. Patients scheduled for elective VATS lobectomy/segmentectomy will be screened and recruited. The researchers will explain the clinical trial and obtain informed consent from eligible patients on the day prior to surgery. The participant timeline is presented in Table 1.

**Table 1:** Schedule of assessments

	Enrolment	Allocation	Primary study period				Follow-up				
Timepoint	−1d	0	PRE: 0–30 min	OLV for 30 min	5 min after the end of OLV	PACU	POD 1	POD 2	POD 3–7	POD 30	POD 60
**Enrolments**											
Eligibility screen	×										
Informed consent	×										
Allocation		×									
**Interventions**											
TPVB			×								
**Assessments**											
Baseline information											
Composite outcome of PPCs							×	×	×	×	×
LUS score	×						×	×			
Blood gas analysis	×			×	×	×					
NRS score	×						×	×		×	×
QoR-15 score	×						×	×		×	×
Complications assessment						×	×	×	×	×	×
Mortality rate										×	×

*PRE* preoperative, *TPVB* thoracic paravertebral block, *PPCs* postoperative pulmonary complications, *OLV* one-lung ventilation, *PACU* post anaesthesia care unit, *POD* postoperative day, *LUS* lung ultrasound, *NRS* numeric rating scale, *QoR-15* quality of recovery with the 15-item scale

### Inclusion criteria

1. Patients scheduled for elective VATS lobectomy/segmentectomy with an expected operation duration (from skin incision to suture) longer than 1 h;
2. Age > 18 years; and
3. ASA: I–III.

### Exclusion criteria

1. Patients with acute or chronic respiratory failure, chronic obstructive pulmonary disease GOLD grade ≥ III, poorly controlled asthma or acute respiratory distress syndrome (ARDS, according to the new definition of ARDS proposed at the 2011 Berlin Conference);
2. Severe cardiovascular complications (defined as NYHA grade IV, acute coronary syndrome, or persistent ventricular tachycardia);
3. A history of ipsilateral thoracotomy or placed under mechanical ventilation within 4 weeks prior to surgery;
4. Patients with contraindications to TPVB (coagulation dysfunction, anticoagulation or antiplatelet therapy, skin ulcer infection, local anaesthetic allergy, spinal deformity, etc.);
5. Patients with trachea malformation or who underwent tracheotomy; and
6. Pregnant or lactating patients.

The experimental design does not involve healthy volunteers or vulnerable groups.

### Randomization and blinding

Stratified blocked randomization will be used in this study. Stratification is performed differently at different centres. To ensure that the results of each centre are consistent and comparable, the Beijing Tongren Hospital affiliated with Capital Medical University will include 152 participants, and the Beijing Chest Hospital affiliated with Capital Medical University will include 150 participants. Within each centre, participants will be randomized based on enrolment time. The blocks will be divided into two groups at a 1:1 ratio using a random table method. The allocation scheme between blocks will also be randomized. Packet information will be hidden in serially numbered, sealed and opaque envelopes for allocation hiding.

Due to the different anaesthesia methods planned to be implemented in this study, the operators cannot be blinded. Participant information will not be known to the follow-up researchers, outcome assessors, data collectors, members of the radiology expert panel or statisticians. If a major adverse event that poses a serious threat to the safety of the subject occurs during the perioperative period and the anaesthesiologist or attending physician deems it necessary to know the subject’s grouping, an authorized blind person may open an emergency blind letter, record the time and cause of the event and name of the blind person, and immediately report it to the supervisory board.

### Preparation before operation

Chest computed tomography scans, NRS scores and LUS scores will be obtained for all patients before surgery. Researchers will guide them on how to self-assess the severity of pain using the numerical Rating Scale (NRS). All patients will fast from food and water for 8 h and 2 h before surgery, respectively.

Upon arrival to the operating room, a peripheral vein of the upper limb will be opened, and sodium lactate Ringer solution will be gradually infused. Electrocardiogram and pulse oxygen saturation will be monitored, radial artery puncture will be performed under local anaesthesia, invasive arterial blood pressure will be monitored, and arterial blood gas analysis will be performed without oxygen as the basic value. The patient will receive oxygen through a mask at a flow rate of 5 L/min. Midazolam 0.02 mg/kg and sufentanil 0.1 μg/kg will be administered intravenously for sedation and analgesia, respectively.

### Intervention

Patients randomly assigned to the PV group (GA combined with TPVB) will be placed in the lateral position and punctured using the out-of-plane technique guided by ultrasound (Shenzhen Huasheng Navi low-frequency convex array probe). Fifteen millilitres of 0.5% ropivacaine will be administered to the T4 and T7 thoracic paravertebral spaces for a total of 30 ml. After injection, the pleura of the punctured segment and adjacent segments should be obviously moved down. For successful puncture, the patient will be placed in the supine position. After 5, 10, and 15 min, the range of sensory blockage was tested at T3~T8 to cover the surgical area. Patients in the C group will not undergo any nerve block.

### Anaesthesia management

#### Induction and maintenance of general anaesthesia

Before anaesthesia induction, 0.5 mg atropine and 40 mg prednisone methylprednisolone will be administered to both groups of patients, followed by intravenous anaesthesia induction with 0.02 mg/kg midazolam, 0.3–0.4 μg/kg sufentanil, 0.2–0.3 mg/kg etomidate, 1–2 mg/kg propofol and 0.6 mg/kg rocuronium. Anaesthesia depth will be maintained by a combination of intravenous and inhalation anaesthesia (inhalation of sevoflurane 0.7–1.0 MAC [17] and continuous infusion of propofol 4–6 mg/kg/h) to maintain a bispectral index monitor range of 40–60. For intraoperative analgesia, remifentanil will be continuously pumped at 0.1–0.2 mg/kg/min, and sufentanil will be intermittently injected intravenously. During the operation, the drug dose will be dynamically adjusted according to the changes in arterial blood pressure and heart rate. If necessary, noradrenaline will be pumped to maintain stable blood pressure. According to muscle relaxation monitoring, rocuronium 0.1 mg/kg will be added (maintain T1 = 0, TOF ratio = 0) to ensure deep muscle relaxation during the operation. As soon as the thoracic incision is closed, all patients will receive another intravenous drip of sufentanil 5 mg and ondansetron 8 mg. Under the guidance of neuromuscular function monitoring, the patients will be given a muscle relaxant antagonist (neostigmine 0.04 mg/kg + atropine 0.02 mg/kg) when T2 appeared. When the TOF ratio is > 90% and the tidal volume (V_T_) is > 6 ml/kg of the predicted body weight (PBW), the endotracheal tube will be removed.

#### Lung protection ventilation management plan

One hundred per cent fraction of inspired oxygen (FiO_2_) will be used for preoxygenation before induction, and 100% FiO_2_ will be used for ventilation before OLV. After placement of the double-lumen bronchial tube under the visual laryngoscope and positioning of the fiberoptic bronchoscope, a manual recruitment manoeuvre (RM) will be performed.

Two-ventilation management (TLV): V_T_ 8 ml/kg PBW, PEEP 5 cmH_2_O, FiO_2_ 100%, volume-controlled ventilation (VCV) mode, end-inspiratory pause (EIP) 10%, inspiratory: expiratory (I:E) 1:2, RR 12 breaths/min. OLV management: V_T_ 6 ml/kg PBW, PEEP 5 cmH_2_O, RR 14 breaths/min, VCV mode, EIP 10%, I:E 1:2 as initial settings [18]. At the beginning of OLV, 60% FiO_2_ will be used until the peak airway pressure is less than 30 cmH_2_O and the airway plateau pressure is less than 20 cmH_2_O [19]. RM will be performed every 30 min (RM is performed at the request of the operator or when the patient's trachea tube is disconnected from the ventilator and retime).

Following OLV, lung recruitment will be performed, followed by TLV and protective ventilation. The pressure will be slowly increased to 30 cmH_2_O and maintained for 30 s [20]. As a prerequisite to lung recruitment, a patient must have haemodynamic stability, which means a systolic blood pressure ≥ 90 mmHg or within ± 30% of the preoperative value, a heart rate 60–100 beats/min, and stroke volume variation < 13%. In cases of haemodynamic instability, new arrhythmia or pulse oxygen saturation (SpO_2_) < 90% in the process of lung recruitment will be stopped immediately.

#### Intraoperative fluid management

To prevent the increase in intrapulmonary shunt and pulmonary oedema caused by excessive infusion, the infusion volume should be controlled during the operation, mainly to maintain and supplement fluid loss, and the crystalloid solution volume should be kept below 6 ml kg^−1^ h^−1^ during the operation [21]. The crystalloid solution:colloidal solution ratio should be 2:1 (the blood loss should be calculated accurately during the operation). The volume, character and composition of postoperative drainage fluid after the operation must be considered.)

#### Temperature monitoring and insulation

To maintain a constant nasopharyngeal temperature and to reduce the effect of hypothermia on hypoxic pulmonary vasoconstriction, methods such as controlling the ambient temperature of the operating room, using heating blankets, heating infusion fluids, and heating flushing fluids will be used.

#### Postoperative management

After the operation, the patient-controlled intravenous analgesia device will contain sufentanil 1.5 μg/kg + ondansetron 24 mg diluted to 100 ml, the background dose will be 1 ml/h, the single dose will be 2 ml, and the locking time will be set to 15 min. Post Anaesthesia Care Unit (PACU) rescue analgesia: when the patient's NRS score is 4–6, flurbiprofen axetil 50 mg will be injected intravenously each time for 1 min; when the patient’s NRS score is ≥ 7, sufentanil 2.5 μg will be injected intravenously each time [16]. Patients will be encouraged to perform activities and deep breathing exercises early after surgery.

### Endpoint

#### Primary endpoint indicators and definitions

For the composite outcome of PPCs within 7 days after surgery, the weight of each complication was equal. Patients with at least one complication will be considered to have experienced the primary endpoint. Imaging data will be interpreted by members of the radiology expert panel. PPCs include:

A. Pneumonia: When a patient receives antibiotics for a suspected respiratory infection and meets at least one of the following criteria: new or changed sputum, new or changed lung opacities, fever (tympanic temperature > 38 °C), leukocyte count > 12×10^9^/L;
B. Aspiration pneumonitis: Acute lung injury after the inhalation of regurgitated gastric contents;
C. Atelectasis: Lung opacification with a shift of the mediastinum, hilum or hemidiaphragm towards the affected area and compensatory overinflation in the adjacent nonatelectatic lung;
D. Respiratory failure: When postoperative arterial oxygen partial pressure (PaO_2_) <60 mmHg on room air, a ratio PaO_2_ to inspired oxygen fraction <300 or arterial oxyhaemoglobin saturation measured with pulse oximetry <90% and requiring oxygen therapy;
E. Bronchospasm: Newly detected expiratory wheezing treated with bronchodilators;
F. Pulmonary congestion: Clinical signs of congestion, such as dyspnoea, oedema, rales, and jugular venous distention, with or without chest X-ray demonstrating an increase in vascular markings and diffuse alveolar interstitial infiltrates;
G. Pleural effusion: Chest X-ray demonstrating blunting of the costophrenic angle, loss of the sharp silhouette of the ipsilateral hemidiaphragm in the upright position, evidence of displacement of adjacent anatomical structures, or (in the supine position) a hazy opacity on one hemithorax with preserved vascular shadows; and
H. Pneumothorax: Air in the pleural space with no vascular bed surrounding the visceral pleura.

#### Secondary endpoint indicators and definitions

A. Blood gas analysis: Arterial blood taken at the place of radial artery catheterization before, during and after the operation for blood gas analysis, and arterial blood pH, PaO_2_, arterial carbon dioxide pressure (PaCO_2_), concentration of arterial blood lactate, and oxygenation index will be recorded.
B. LUS score: Bedside pulmonary ultrasound will be performed by professional sonographers trained to obtain uniform standards before, 24 h after, and 48 h after surgery. Posture: To ensure the consistency of the preoperative and postoperative examination positions, the end sitting position will be used for pulmonary ultrasound; Ultrasonic selection: Shenzhen Huasheng Navi low-frequency convex array probe; Operation method: Each side of the chest of the patient will be divided into six regions, with the front axillary line, the posterior axillary line, and the nipple line as boundaries. The ultrasonic probe will be used to scan each area from right to left, from top to bottom, and from front to back. Score according to the number of B lines in the LUS image of each area. 0: B lines ≤ 2; 1 point: > 2 well-spaced B-lines; 2 points: Multiple coalescent B lines; 3 points: white lung (lung consolidation);
C. The incidence of various PPCs: pneumonia; aspiration pneumonitis; atelectasis; respiratory failure; bronchospasm; pulmonary congestion; pleural effusion; pneumothorax within 7 days after surgery.
D. The incidence of various postoperative extrapulmonary complications including arrhythmia; cardiovascular complications (arrhythmias, acute coronary syndrome, myocardial infarction, acute congestive heart failure); cerebrovascular complications (cerebral infarction, cerebral haemorrhage); postoperative cognitive dysfunction; postoperative renal complications; shock; and postoperative extrapulmonary infection within 7 days after surgery.
E. Evaluation of postoperative analgesia: including chest NRS scores during periods of rest and coughing after surgery (Participants will be asked to rate their average pain intensity during periods of rest and coughing by selecting a single number from 0 to 10. NRS scores range from 0, indicating “No pain”, to 10, indicating “The most intense pain imaginable”. The higher the score, the more severe the pain; the postoperative quality of recovery according to the 15-item score (QoR-15, a global indicator for evaluating postoperative recovery, assesses patient recovery quality from five dimensions: physical comfort, physical independence, emotional state, psychological support and pain. The total score ranges from 0 [poorest recovery quality] to 150 [best recovery quality]. The total sufentanil consumption 48 h after surgery and the incidence of opioid-related adverse effects will also be recorded;
F. Hospitalization-related indicators: unplanned ICU hospitalization rate and duration, postoperative hospitalization duration, and hospitalization expense;
G. Long-term prognosis indicators include the incidences of pulmonary and extrapulmonary complications, NRS score, QoR-15 score and mortality rate at 1 and 3 months after operation.

#### General information

Age, sex, height, weight, body mass index, preoperative complications, American Society of Anaesthesiologists classification (ASA) classification, ARISCAT score, preoperative pulmonary function, surgical history, SpO_2_ (≥ 96%, 91–95%, ≤ 90%), one-month respiratory infection, NYHA heart function classification, preoperative medication history, etc.

#### Intraoperative information

Name of operation, duration of operation, time of anaesthesia, amount of fluids, duration of ventilation in one lung, body temperature, adverse reaction of operation, use of vasoactive drugs, etc.

### RESCUE

#### Treatment of hypoxaemia (SpO2 ≤ 92%, lasting > 1 min) during OLV

A. First, eliminate diseases that cause increased airway resistance, pneumothorax, unstable haemodynamics (to avoid drop in blood pressure caused by operator compression of the inferior vena cava and TPVB), ventilator failure, etc., and catheter displacement due to fibrobronchoscopy;
B. Gradually increase FiO_2_ to 100% (Table 2);
C. In the ventilatory lung subjected to RM, the PEEP level will be gradually increased to 10 cmH_2_O (Table 2);

**Table 2:** Treatment steps for hypoxaemia

	FiO_2_	PEEP
Step1	0.6	5
Step2	0.7	5
Step…	…	5
Step5	1.0	5
Step6	1.0	RM+6
Step7	1.0	RM+7
Step…	1.0	RM+…
Step10	1.0	RM+10

*FiO*_*2*_ fraction of inspired oxygen, *PEEP* positive end-expiratory pressure, *RM* recruitment manoeuvres

D. Inhaled oxygen to the unventilated lungs: 3 L/min [11], inhaled continuous positive airway pressure (CPAP) if necessary in the nonventilatory lung [22];
E. If it still fails to meet the standard, it should be adjusted to TLV and RM (this measure can be taken directly in cases of severe and sudden decreases in SpO_2_); and
F. In any rescue step, if the SpO_2_ of a patient with stable haemodynamics worsens further, the anaesthesiologist can consider reducing the PEEP and increasing the V_T_ to 8 ml/kg PBW.

#### Treatment of severe hypercapnia during OLV (blood gas analysis PaCO2 > 50 mmHg)

A. Gradually increase RR to 20 breaths/min (if peak airway pressure ≥ 30 cmH_2_O, the PEEP can be reduced);
B. Gradually increase the V_T_ to 8 ml/kg PBW; and
C. Switch to TLV and perform RM.

#### When any of the following clinical conditions occur, PEEP can be modified according to the judgement of the anaesthesiologist

A. Systolic blood pressure < 90 mmHg or decrease by more than 30% of the base value, and the use of liquid therapy and/or the administration of vasoactive drugs has not yet increased to 90 mmHg;
B. In the case of blood loss, Hb > 70 g/L can only be maintained through massive blood transfusion (definition of massive blood transfusion: 24 h blood transfusion volume is greater than 100% of blood volume or 4 h blood transfusion volume is greater than 50% of blood volume); and
C. Any other life-threatening complications occurring during the operation, and patients with PEEP changes can benefit.

#### Rescue in the PACU

A. In the case of arterial hypoxaemia (SpO_2_ < 92%) or hypercapnia (pH < 7.30 and PaCO_2_ > 50 mmHg) and tachypnea (RR > 25 breaths/min) after extubation, after excluding laryngeal spasm, bronchospasm, pneumothorax or haemodynamics, the patient can be given a mask to pressurize oxygen and undergo noninvasive ventilator ventilation.
B. If the patient has haemodynamic instability, electrocardiogram signs of myocardial ischaemia or malignant arrhythmia, or a GCS < 9.4, and needs sedatives, he or she should be intubated again to assist breathing as appropriate.

#### Perioperative treatment of other adverse events

Other adverse occurrences must be managed in accordance with clinical best practices; if the researcher does not think a subject's involvement is in their best interests, they have the right to end it at any time. The CRF will contain a record of the circumstances and reasons for terminating the trial. Patients have the right to withdraw from the study at any stage and at any time without affecting their medical treatment and rights.

#### Provisions for posttrial care

Participants will have access to study clinics for posttrial care through the routine health system.

### Data management

During the study period, the information of all the patients will be continuously monitored, including anaesthesia records, hospitalization records, daily visits during hospitalization and daily telephone visits during discharge follow-up. Baseline data for participants will be collected preoperatively, intraoperative data will be collected by a researcher who will not know the group of participants, laboratory tests will be performed in the laboratory department, and imaging will be performed by radiologists and sonographers. All relevant data will be stored in the study folder and will only be accessible to the study group. All serious adverse, unexpected or possibly related events will be recorded in the CRF by trained researchers.

Before the start of the study, uniform standard operating procedures will be developed, and effective training will be provided to researchers, doctors, and nurses in each centre. During the patient's hospitalization and one month and three months after surgery, the investigator will follow up with the participants and provide diagnosis and treatment recommendations to improve patient compliance.

The data that will be entered into the database will not include the subject's name or other personal information, and only the unique code identification corresponding to the subject's name will be entered. The results of the study to be published will contain only the information in the database. To avoid human errors in entry, two data entry personnel will be used to enter data independently and synchronously, and then the machine will compare the data to extract duplicates. No interim analysis has been designed for this study.

### Oversight and monitoring

An anaesthesiologist, a sonographer, and a radiologist were employed at the coordination centre for this study. Two statisticians will guide the statistical analysis of the data obtained for this study. The data monitoring committee (DMC) consists of an anaesthesiologist, an ethics expert, a radiologist, a statistician, and a clinical trial management expert. All team members are independent of the sponsor, and there are no conflicts of interest. The DMC will hold a monthly meeting to review research progress, check data integrity, and monitor the occurrence of serious adverse events (SAEs).

When a serious adverse event occurs, the researcher will immediately report it to the DMC, expert group, and ethics committee. Researchers will record the symptoms, severity, duration, relevant medical measures taken, outcome, and correlation with intervention measures in the CRF form and submit a complete SAE report form to the research team within 24 h. Researchers will follow up on SAEs until the adverse event disappears, returns to baseline level, or is lost.

### Statistical analysis

#### Sample size calculation

PASS 15.0 was used to compute the sample size. The incidence of PPCs after surgery was 48% in the C group and 32% in the PV group, according to a pilot study [18] (the pilot study sample included 50 patients). The difference test of the rate between the two groups was selected, α: 0.05, 1-β: 0.8. The estimated sample size for the bilateral test was 144 cases in each group. The sample size was increased to 151 in each group for a total of 302 cases, accounting for the 5% probability of loss to follow-up.

#### Statistical method

As long as the participants are enrolled, even if they have only gone through a partial trial procedure, they will still be included in the statistical analysis in accordance with the intention-to-treat analysis principle. Moreover, the per-protocol analysis will be used as a sensitivity analysis. We expect very few patients to be lost to follow-up during the hospital stay. As a result, missing data will be minimized when analysing primary outcomes. If statistical methods are needed to interpret missing data for secondary outcomes, multiple imputation will be used. No interim analysis has been designed for this study.

SPSS 25.0 will be used to process the data. The measurement data with a normal distribution will be expressed by ($\overline{x}$± *s*), and the independent sample *t* test will be used for comparison between groups. The measurement data with a nonnormal distribution will be expressed by M (Q1, Q3), and *the Mann–Whitney U* test will be adopted. Counting data will be described by the number of cases (%) and compared between groups by *χ*^2^ inspection. The rank sum test will be used for grade data. The interaction of covariates will be analysed by logistic regression to evaluate the risk factors for each subgroup, and a *P* value < 0.05 will be considered statistically significant.

## Discussion

The objective of this prospective, double-centre, randomized controlled trial of patients undergoing VATS pulmonary surgery is to determine whether TPVB can reduce the incidence of PPCs in patients and to evaluate the clinical parameters related to lung injury, postoperative analgesia indicators, and long-term prognosis indicators. This topic is very important because reducing the incidence of PPCs can improve the short-term and long-term prognosis of patients after surgery, reduce the mortality rate, and lower medical costs. During VATS, the application of OLV technology and the occurrence of perioperative acute pain will increase the risk of PPCs such as atelectasis and pneumonia, which is not conducive to the rapid recovery of patients [9]. PPCs often manifest as a composite outcome, which can increase the early postoperative mortality rate and prolong the hospital stay, mainly within seven days after surgery [17]. Therefore, this study will monitor the incidence of PPCs within 7 days in subjects after surgery while paying attention to the occurrence and outcome of long-term. complications. Sufficient postoperative analgesia has significant benefits in improving postoperative lung function and reducing the occurrence of early postoperative adverse events in patients [23]. TEA combined with GA is considered superior to opioid-based pain management alone and has been proven to improve postoperative lung function, reduce the incidence of lung complications such as pneumonia, and reduce the perioperative mortality rate [11, 12, 24]. However, TEA blocks bilateral sympathetic and intercostal nerves, resulting in vasodilation and intercostal muscle weakness, which may have an impact on the respiratory and circulatory systems in the perioperative period [9]. As an important means of multimodal analgesia, peripheral nerve block is being increasingly used to control acute pain after VATS. The analgesic effect of TPVB is similar to that of TEA [25], with a lower failure rate and fewer complications, such as intraspinal haematoma, hypotension, nausea, and urinary retention, than TEA [9, 26]. Shu-Qing Zhen et al. found that when GA combined with TPVB was used in radical lung tumour resection, the postoperative levels of adrenaline, norepinephrine, dopamine, TNF-α and IL-6 were significantly reduced after surgery, which proved that TPVB could also inhibit the stress response and inflammatory response during surgery, reduce the risk of pneumonia injury, and effectively protect the lung. TPVB under ultrasound guidance can be used to further ensure an analgesic effect by observing the enlargement of the low echo area and the anterior displacement of the pleura under direct ultrasound during the injection of the drug.

Needle insertion using an out-of-plane technique further reduces the risks of central block and spinal cord injury [27]. However, this method requires the needle to be angled towards the ultrasound plane, thus reducing the visibility of the needle. Therefore, we will use echogenic needles to enhance the development effect and make the needle tip easier to position. We will perform two single punctures in the T4 and T7 thoracic paravertebral space on the affected side of the subject to guarantee the effect of analgesia on the plane selected for surgery [28]. It has been reported that continuous paravertebral block is achieved by inserting a catheter into the paravertebral space. However, a single injection of TPVB will be administered in this study. There is no exact way to know the precise location of the catheter tip after catheter placement. A study found that although the catheter did not change the skin, the subcutaneous tissue and muscles surrounding the catheter changed when the patient's position changed, causing the catheter tip to shift and greatly affecting the extent of diffusion of the drug injected from the catheter [29]. To prolong the duration of postoperative analgesia, a patient-controlled intravenous analgesic device will be used in combination with analgesia. In summary, this study will better evaluate the impact of the use of TPVB on the incidence of PPCs and the long-term prognosis in patients undergoing VATS pulmonary surgery. The results of this research will be disseminated in its entirety in international peer-reviewed journals. The results may provide clinical evidence supporting the optimization of perioperative lung protection strategies.

## Trial status

Protocol version number V2.0/March 10, 2023.

Date recruitment started, 1 July 2023.

Date of anticipated completion, 31 December 2023.

### Supplementary Information

**Additional file 1.** SPIRIT 2013 checklist.

## Abbreviations

ARDS: Acute respiratory distress syndrome
ARISCAT: Assess Respiratory Risk in Surgical Patients in Catalonia
ASA: American Society of Anaesthesiologists Classification
CPAP: Continuous positive airway pressure
DMC: Data monitoring committee
EIP: End-inspiratory pause
FiO_2_: Fraction of inspired oxygen
GA: General anaesthesia
NRS: Numerical Rating Scale
OLV: One-lung ventilation
PaCO_2_: Arterial carbon dioxide pressure
PACU: Post anaesthesia care unit
PaO_2_: Arterial oxygen partial pressure
PBW: predicted body weight
PEEP: Positive end-expiratory pressure
PPCs: Postoperative pulmonary complications
QoR-15: Quality of recovery with the 15-item score
RM: Recruitment manoeuvres
SAE: Serious adverse events
SpO_2_: Pulse oxygen saturation
TEA: Thoracic epidural anaesthetic
TLV: Two-ventilation management
TOF: Train of four stimulation
TPVB: Thoracic paravertebral block
VATS: Video-assisted thoracoscopic surgery
VCV: Volume-controlled ventilation
